# Fire ant-venom anaphylaxis prevalence in the general population and patients with systemic mastocytosis

**DOI:** 10.3389/falgy.2025.1570123

**Published:** 2025-03-31

**Authors:** Jeremy C. McMurray, Brandon J. Schornack, Karla E. Adams, Robert L. McCoy, Amanda K. Marshall, Janet A. Brunader, Irina Maric, Dean D. Metcalfe, Nathan A. Boggs

**Affiliations:** ^1^ Allergy & Immunology Service, Walter Reed National Military Medical Center, Bethesda, MD, United States; ^2^ Department of Pediatrics, Uniformed Services University, Bethesda, MD, United States; ^3^ Allergy & Immunology Service, Wilford Hall Ambulatory Surgical Center, San Antonio, TX, United States; ^4^ The United States Army Centralized Allergen Extract Laboratory (USACAEL), Silver Spring, MD, United States; ^5^ Defense Health Agency, Defense Healthcare Management Systems, Falls Church, VA, United States; ^6^ Defense Health Agency Immunization Healthcare Division, Falls Church, VA, United States; ^7^ Hematology Section, Department of Laboratory Medicine, National Institutes of Health, Bethesda, MD, United States; ^8^ Laboratory of Allergic Diseases, National Institute of Allergy and Infectious Diseases, National Institutes of Health, Bethesda, MD, United States; ^9^ Department of Medicine, Uniformed Services University, Bethesda, MD, United States

**Keywords:** red and black fire ants, *Solenopsis invicta* and *richteri*, fire ant-venom anaphylaxis prevalence, flying hymenoptera-venom anaphylaxis prevalence, systemic mastocytosis (SM)

## Abstract

**Background:**

Stinging Hymenoptera can induce fatal anaphylaxis, especially in patients with systemic mastocytosis. Fire ants, *Solenopsis invicta* and *S. richteri,* from South America have recently colonized three continents. Prevalence of fire ant-venom anaphylaxis in the general population and in systemic mastocytosis is unknown. The aim was to determine fire ant-venom anaphylaxis prevalence among Tricare beneficiaries and those with systemic mastocytosis.

**Methods:**

We queried the beneficiary immunotherapy prescription database for patients who received immunotherapy with Hymenoptera venom or fire ant whole-body extract and the Tricare beneficiary population health registry database for patients with an ICD−10 code for Hymenoptera venom allergy (HVA). Greater than 95% of the beneficiary population were patients living in the United States. Chart review of a random sample of 150 patients linked to a HVA ICD-10 code was performed to determine the percent of patients with Hymenoptera-venom anaphylaxis. Retrospective review of a systemic mastocytosis cohort was performed to assess fire ant-venom anaphylaxis rate and treatment patterns.

**Results:**

Fire ant immunotherapy was the most frequently ordered individual immunotherapy prescription 45.9% (*n* = 878). Fire ant prescriptions surpassed all flying Hymenoptera immunotherapy prescriptions combined in six states. Fire ant and flying Hymenoptera-venom anaphylaxis prevalence in the general population was 0.048% and 0.083%, respectively. Fire ant-venom anaphylaxis prevalence in the 14 colonized states was 0.085%. More patients with systemic mastocytosis had anaphylaxis triggered by fire ant than all flying Hymenoptera combined.

**Conclusion:**

Fire ant-venom anaphylaxis prevalence in the general population and patients with systemic mastocytosis is higher than all flying Hymenoptera-venom anaphylaxis combined in colonized states. Fire ant-venom anaphylaxis in systemic mastocytosis is frequently misdiagnosed and not treated with epinephrine.

## Introduction

The Hymenoptera order of insects arose 280 million years ago and includes many stinging species that can trigger anaphylaxis in humans. The genera of stinging Hymenoptera responsible for most anaphylaxis include *Apis* (honeybee), *Vespula* (yellow jackets), *Polistes* (wasps), *Dolichovespula* (hornets), and *Solenopsis* (fire ants or FAs). The stinger, an adaptation of the egg-laying ovipositor, evolved in the common ancestor of the Aculeata, a subclade of Hymenoptera, around 142 million years ago (1). Hymenoptera venom proteins evolved before the stinger and were likely co-opted from roles in modulating the environment of a plant host for offspring (2). Hymenoptera venom varies in composition between species and among individuals within a species and is a mixture of amines as well as proteins. FA venoms are unique in having a high content of water-insoluble alkaloids that function as toxins, repellents, and pheromones (3).

Many venom genes and the corresponding proteins are shared between families, although some are unique. For example, the gene encoding hyaluronidase, originally sequenced in honeybee in 1993, is found in all Hymenoptera species (4). The four major proteins present in FA venom and corresponding complementary DNA have also been sequenced. Two of the four FA venom proteins, phospholipase A1 and antigen 5, are also present in vespid venom (5). By contrast, the gene encoding the pain inducing-protein melittin is found only in the bee lineage (4).

The amount of venom protein delivered in a single sting is highest in honey bee (25–150 mcg) and vespids (2–20 mcg) and lowest in FAs (10–100 ng) (6, 7). Despite the small protein content, the sensitization rate to FA venom can be >90% in colonized regions and is higher than sensitization to other stinging Hymenoptera, likely due to high sting exposure, multiple stings, aggressive insect behavior, and potentially increased venom protein allergenicity (8–11). Notably, immunotherapy (IT) with insect venom has been utilized successfully for many decades to prevent anaphylaxis in those with Hymenoptera venom allergy (HVA) (12, 13). Similarly, whole-body extract IT (WBE-IT) is effective in the treatment of FA-venom anaphylaxis (14, 15).

Anaphylaxis following envenomation can be fatal with an estimated 60–100 deaths per year in the United States (U.S.) from honeybee, yellow jacket, hornet, and wasp (referred to as flying Hymenoptera or FH) (16–24). Systemic mastocytosis (SM) is a major risk factor for severe and fatal anaphylaxis to FH (25–35). SM, as defined by the World Health Organization (WHO), is a rare myeloid neoplasm where there is abnormal growth and activation of neoplastic mast cells (36). The severe anaphylaxis triggered by FH in patients with SM is generally characterized by cardiovascular collapse in the absence of urticaria and angioedema (37). Approximately half of patients with SM have been reported to have anaphylaxis triggered by FH. Yet, only 11% of anaphylaxis events in patients with SM are treated with epinephrine despite delays in epinephrine administration being linked to death (35, 38). FH IT is recommended lifelong in patients with SM due to persistent risk after the typical multi-year course of treatment (33, 39). Patients with FH anaphylaxis are screened for SM by a) physical exam to identify monomorphic lesions of maculopapular cutaneous mastocytosis, b) calculation of a symptom- and basal serum tryptase (BST)-based risk scoring tool, and c) by assessment for BST discordance with tryptase genotype (40).

Several studies have sought to estimate the prevalence of FH-venom anaphylaxis in the U.S. and Europe. The prevalence of FH-venom allergy in children enrolled in scouting programs was first estimated in the early 1970s to be 0.38%–0.80% based upon surveys conducted from parents of nearly 10,000 children in Rhode Island (41–43). Depending on the population and study method, the prevalence of FH-venom allergy in adults is estimated to be 0.17%–3.3% (44–48).

There are two main species of FAs, *Solenopsis invicta* (red FA) and *S. richteri* (black FA), as well as interspecific hybrids that inhabit the U.S. Both species are thought to have arrived in the U.S. from South America in the early 1900s near Mobile, Alabama, and subsequently, the habitat of *S. invicta*, has expanded to 14 states and Puerto Rico. In a 1989 survey, physicians reported treating 20,755 patients for reactions to FAs, of which 2% included treatment for anaphylaxis (49). Yet, the prevalence of FA-venom anaphylaxis in the general population and in patients with SM remains to be carefully assessed. Here, we present an estimate of the prevalence of FA-venom anaphylaxis in the general population of Tricare beneficiaries and within the subset of patients with SM.

## Materials and methods

### Venom immunotherapy prescription database

The Military Health System (MHS) is a U.S. government-managed medical system for Tricare beneficiaries including active-duty service members (ADSMs), retirees, and dependents. Beneficiaries are enrolled into direct care markets operated by the Defense Health Agency or deferred into private sector care. ADSMs comprise 36%, and retirees and dependents comprise 64% of the direct care beneficiary population. The decision to enroll into direct care vs. a private sector network is typically based on geographical distance. Patients may switch between direct care and private sector care over time. Direct care in the MHS is comprised of 20 large markets, 17 small markets, and many stand-alone clinics dispersed throughout the U.S.; as well as 1 large market in the Indo-Pacific and 1 large market in Europe. There were 9,370,300 Tricare beneficiaries total, 2,835,948 beneficiaries enrolled in direct care, and 1,045,565 ADSMs in direct care on December 31, 2023. There is no cost for IT for Tricare beneficiaries.

The U.S. Army Centralized Allergen Extract Laboratory (USACAEL) provides IT with FH venom and FA WBE prescriptions for all Tricare beneficiaries. Individual IT prescriptions include honeybee (HB), yellow jacket (YJ), white-faced hornet (WFH), yellow hornet (YH), and wasp (W) extract orders. A mixed vespid (MV) extract is also available which is a mixture of YJ, WFH, and YH venoms. FA prescriptions are composed of WBE of a single species (red or black FA) or an extract that includes a mix of both FA species. IT prescription ordering privileges are restricted to allergy and immunology physicians and physician assistants. USACAEL has maintained a centralized database of IT prescriptions ordered for Tricare beneficiaries enrolled in direct care since 1990. The beneficiary IT prescription database was queried on January 8, 2024 for a list of all prescriptions containing FA, HB, YJ, WFH, YH, MV, and W from January 1, 1990 to December 31, 2023. Data included patient identifiers, beneficiary status (active, retired, or dependent), the initial and refill IT prescriptions, prescription contents, prescription dates, ordering clinician name, and location of ordering facility (city, state, and zip code).

### Tricare beneficiary population health registry database

The Tricare beneficiary population health registry is a database of current beneficiaries that is linked to the electronic medical record (EMR) and claims data. A query of the database was performed on January 2, 2024 to identify current beneficiaries who had ever been linked to one or more of the following venom allergy ICD-10 codes: T63.44, T63.45, T63.46, Z91.030, and Z91.038. Data for each patient included unique identifiers, specific ICD-10 code(s) linked to venom allergy, and location (city, state, and zip code). Accuracy of ICD-10 codes in HVA diagnosis was assessed (see Supplementary Methods). HVA was defined in this study as anaphylaxis and did not include large local or diffuse cutaneous reactions.

### Systemic mastocytosis patient cohort

The cohort of current Tricare beneficiaries with SM was obtained by querying a previously described mastocytosis registry linked to the Tricare beneficiary population health registry database (40). Individual chart review was performed to determine study eligibility. A patient was included in the study if they had a diagnosis of any subtype of SM or monoclonal mast cell activation syndrome (MMAS) (see Supplementary Methods). Data collected included age, sex, SM subtype, history of monomorphic maculopapular cutaneous mastocytosis (MPCM), *KIT* p.D816 V testing results, BST values, history of anaphylaxis, anaphylaxis triggers, concurrent beta blocker use, concurrent angiotensin converting enzyme inhibitor use, skin test results, specific-IgE test results, history of venom and WBE-IT treatments, anaphylaxis episode counts, anaphylaxis treatments, as well as bone marrow biopsy (BMB) pathology results. Anaphylaxis was defined according to NIAID criteria (See Supplementary Methods). Severity was determined by Ring and Messmer criteria with grades III and IV classified as severe (Supplementary Table 2). Characteristics of the episodes of anaphylaxis collected included the associated trigger, whether the anaphylaxis event was diagnosed correctly at the time it occurred, whether epinephrine was administered, and the total epinephrine dose administered.

Coded data were collected by review of subject charts and recorded into a Microsoft Excel database. Statistical analyses were performed using IBM SPSS v28, and PRISM 10 (GraphPad Software) was used for graphical illustrations. This study was approved by the Walter Reed National Military Medical Center institutional review board.

## Results

### Hymenoptera venom immunotherapy in current Tricare beneficiaries

**Figure 1:**
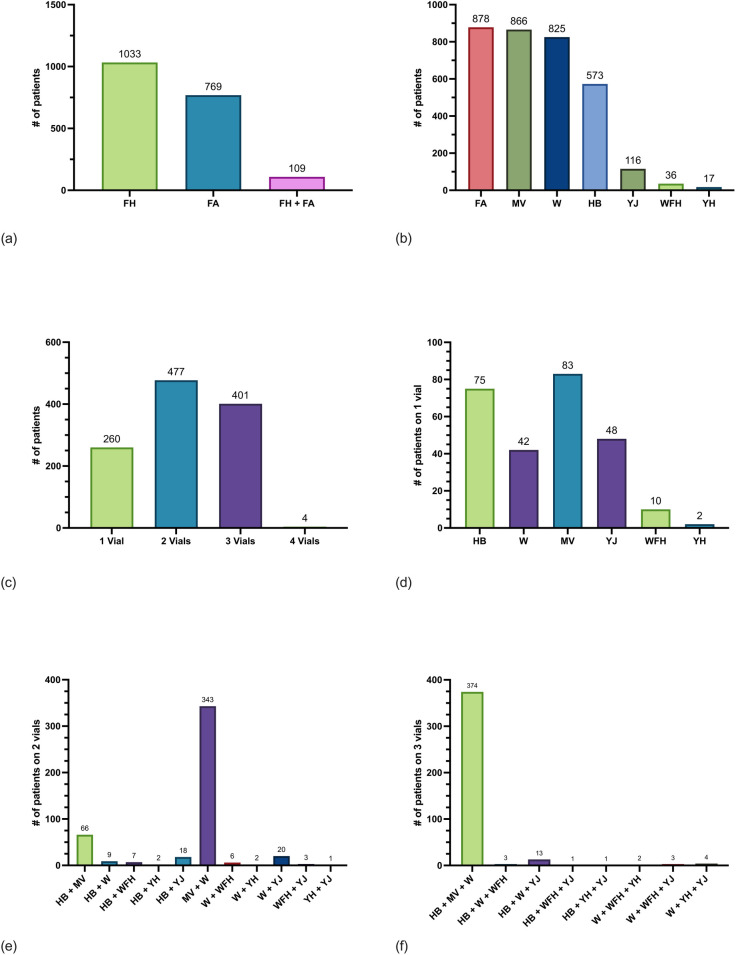
Frequency of current beneficiaries with Hymenoptera IT prescriptions enrolled in direct care. **(a)** Counts of current beneficiaries from an immunotherapy database who were ordered fire ant (FA) whole body extract (WBE), one or more flying Hymenoptera (FH)-venom extracts, or both FA WBE and FH-venom extract. Patients could be counted in only one group. **(b)** Counts of current beneficiaries who were ordered FA WBE, mixed vespid (MV) venom extract, wasp (W) venom extract, honeybee (HB) venom extract, yellow jack (YJ) venom extract, white-faced hornet (WFH) venom extract, and yellow hornet (YH) venom extract. Patients could be counted in more than one group if they were ordered more than one extract. **(c)** Number of patients on treatment with one, two, three, or four vials of FH IT. Vials are composed of one of the following: HB, W, YJ, WFH, YH, or MV venoms. **(d)** Number of patients on one vial only for HB, W, MV, YJ, WFH, and YH venoms. **(e)** Number of patients on two vials for combinations of HB, W, MV, YJ, WFH, and YH venoms. **(f)** Number of patients on three vials for combinations of HB, W, MV, YJ, WFH, and YH venoms. there were four patients on four FH vials (not shown).

A total of 7,237 unique patients enrolled in direct care, private sector care, or the Veteran's Affairs care system had IT prescriptions for HB, FA, YJ, YH, WFH, MV, and/or W ordered in the IT prescription database from January 1, 1990 to December 31, 2023 (Supplementary Figure 1a). Of these 7,237 patients, 26.4% (*n* = 1,911) were current beneficiaries enrolled in direct care on December 31, 2023 with IT prescriptions for FH, FA, or both (Figure 1a). Of this group of current beneficiaries, IT prescriptions were ordered from 89 sites in 31 states in the U.S. as well as 6 sites in 5 countries outside the U.S.

The most frequently ordered IT prescription was FA 45.9% (*n* = 878). The next most frequently ordered IT prescriptions were MV 45.3% (*n* = 866), followed by W 43.2% (*n* = 825), and HB 30.0% (*n* = 573) (Figure 1b). Most FA WBE prescriptions included both *S. invicta* and *S. richteri* 92.1% (*n* = 809) while 7.9% (*n* = 69) contained only *S. invicta* and 0% (*n* = 0) contained only *S. richteri*. Most patients on IT 53.3% (*n* = 1,019) were ordered only one prescription for either HB, FA, MV, WFH, YH, YJ, or W and the remaining patients 46.7% (*n* = 892) were treated with two or more IT prescriptions. A minority of patients 13.6% (*n* = 260) were ordered one FH IT vial only for either HB, YJ, YH, WFH, MV, or W (Figures 1c-f). Of note, 1.0% (*n* = 19) of patients were ordered two of YJ, WFH, and/or YH instead of MV IT.

Notably, 109 patients were treated with both FA WBE-IT and one or more FH IT. Of those 109, 9.2% (*n* = 10) were treated with FA and one additional FH IT vials, 42.2% (*n* = 46) were treated with FA and two other FH IT vials, and 48.6% (*n* = 53) were treated with FA and three other FH IT vials. The FH most frequently ordered with FA was W 86.2% (*n* = 94), followed by MV 85.3% (*n* = 93), HB 55.0% (*n* = 60), YJ 6.4% (*n* = 7), WFH 5.5% (*n* = 6), and YH (*n* = 1).

**Figure 2:**
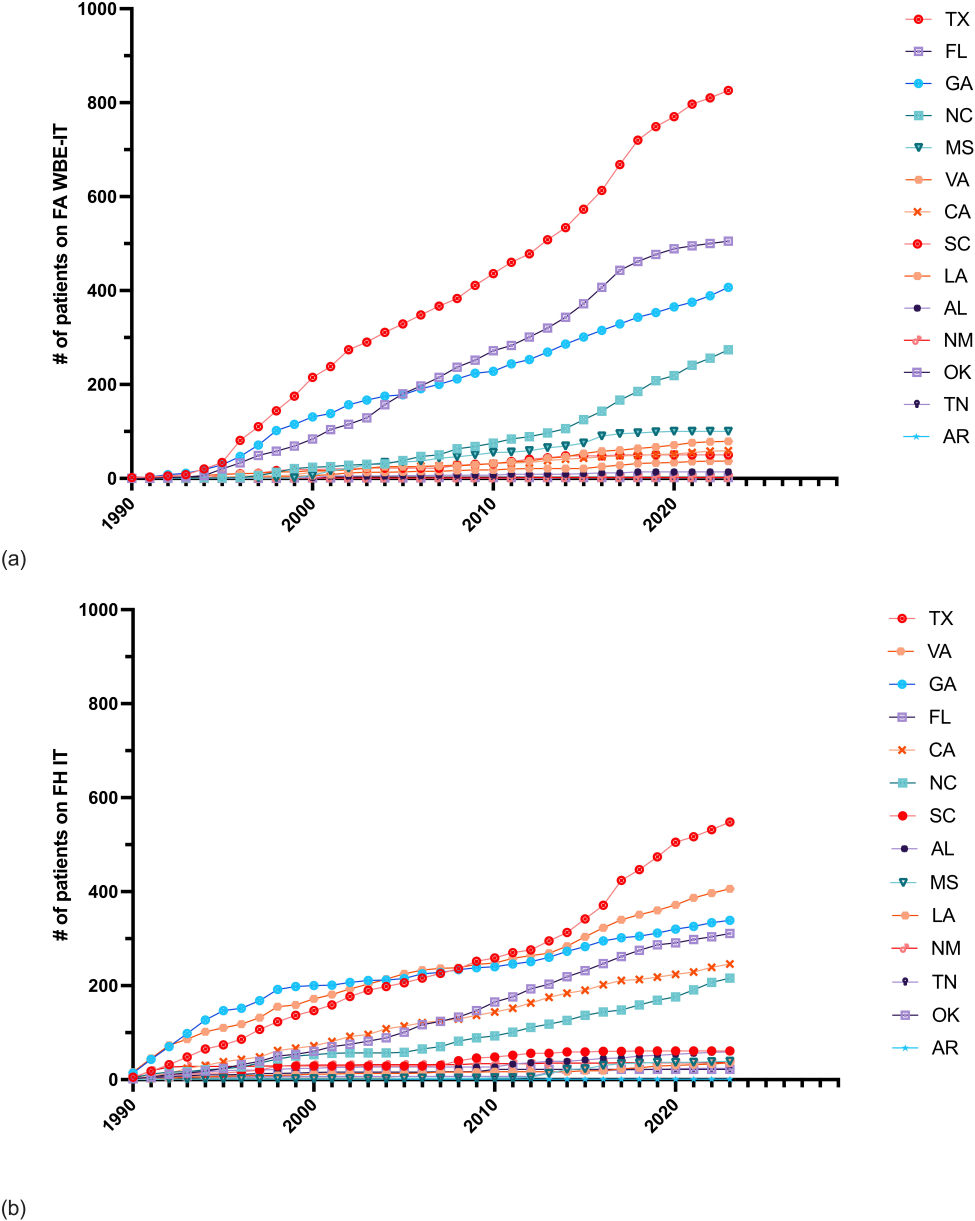
Cumulative counts of initial Hymenoptera IT prescriptions in 14 states colonized by FAs. **(a)** Cumulative annual frequency of patients ordered FA WBE-IT prescriptions by state. **(b)** Cumulative annual frequency of patients ordered any FH IT prescription by state.

FAs have been reported in 14 states in the U.S. and Puerto Rico (Supplementary Table 3). The cumulative number of initial FA WBE-IT prescriptions surpassed the total number of all other initial FH IT combined in six states including Florida by 1996, Texas by 1997, Mississippi by 2000, Georgia by 2012, North Carolina by 2017, and Louisiana by 2022 (Figure 2). By the end of 2023, the total number of initial FA WBE-IT prescriptions remained less than the total number of all other IT prescriptions combined in Virginia, California, South Carolina, Alabama, New Mexico, Tennessee, Oklahoma, and Arkansas.

### Analysis of ICD-10 code usage for HVA

**Figure 3:**
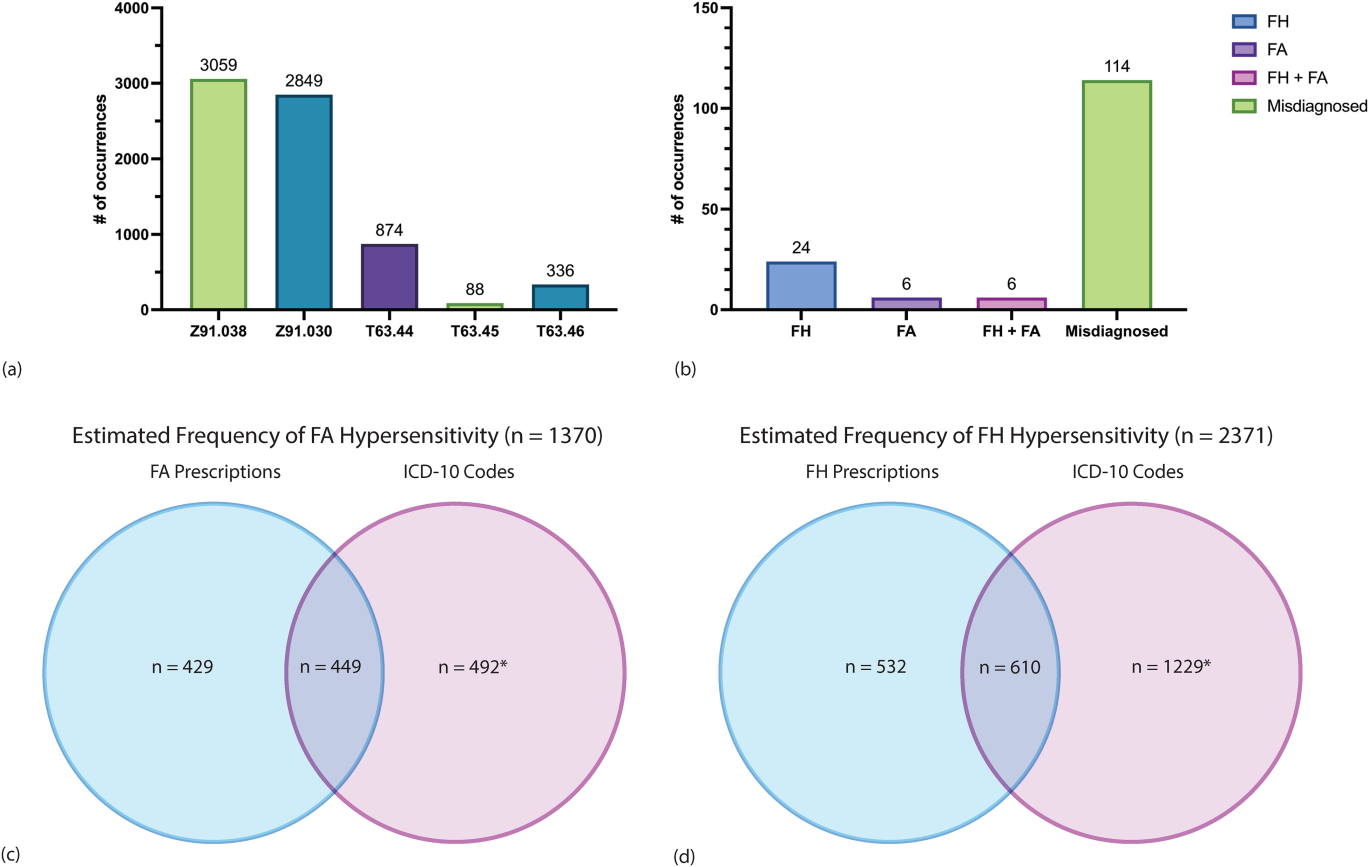
Analysis of ICD-10 code counts among 2,835,948 current Tricare beneficiaries enrolled in direct care. **(a)** Counts of patients assigned one of five ICD-10 codes corresponding to HVA. Each patient is represented once by a single ICD-10 code only. The most recent ICD-10 code was the one assigned to a patient if that patient had more than one linked code to avoid duplicates. Each patient is represented once by a single ICD-10 code only. (See Supplementary Table 1 for description of ICD-10 codes). **(b)** A random sample of 30 patients for each ICD-10 code was obtained and chart review of all 150 patients was performed to determine which patients had the ICD-10 code accurately assigned. Counts of confirmed FH-venom anaphylaxis, FA-venom anaphylaxis, both FH- and FA-venom anaphylaxis, and misdiagnosed cases are shown. **(c)** Estimated counts of FA-venom anaphylaxis through combining FA WBE-IT prescriptions and ICD-10 code data among current beneficiaries. Counts in the blue circle represent patients with any FA WBE-IT prescription who were not represented in the population health registry dataset. Counts in the overlap of the blue and purple circles represent patients with any FA WBE-IT prescription who were also identified in the population health registry dataset. The count in the purple circle was estimated based on multiplying 6,147 by 0.08 and is an estimate of the number of patients with FA-venom anaphylaxis not represented in the IT prescription database. There was an 8.0% true positive rate of FA-venom anaphylaxis as determined by chart review of a random sample of 150 patients with one of the five venom allergy ICD-10 codes from the Tricare beneficiary population health registry dataset. There were 4 patients on FA WBE-IT in the random sample, which would include an estimated additional 164 prescriptions (total 1,042). **(d)** Estimated counts of FH-venom anaphylaxis through combining FH IT prescriptions and ICD-10 code data among current beneficiaries. Counts in the blue circle represent patients with any FH IT prescription who were not represented in the population health registry dataset. Counts in the overlap of the blue and purple circles represent patients with any FH IT prescription who were also identified in the population health registry dataset. The count in the purple circle was estimated based on multiplying 6,147 by 0.2 and is an estimate of the number of patients with FH-venom anaphylaxis not represented in the IT prescription database. There was a 20.0% true positive rate of FH-venom anaphylaxis as determined by chart review of a random sample of 150 patients with one of the five venom allergy ICD-10 codes from the Tricare beneficiary population health registry database. There were 11 patients on FH IT in the random sample, which would include an estimated additional 451 prescriptions (total 1,593).

The Tricare beneficiary population health registry database was queried on January 2, 2024 to identify current beneficiaries in direct care who had ever been linked to one or more of the venom allergy ICD-10 codes; and a total of 7,206 patients were identified (Figure 3a, Supplementary Figure 1b). 449 of the 878 patients with a FA-venom anaphylaxis identified in the IT prescription database were also seen in the Tricare beneficiary population health registry. Similarly, 610 of the patients with FH-venom allergy identified in the IT prescription database were also seen in the population health registry. Fifty-nine patients identified in the IT prescription database who were prescribed both FA and FH IT prescriptions were also identified in population health registry dataset.

Individual chart review was performed on a random sample of 150 patients, 30 of which corresponded to each ICD-10 code, to ascertain the accuracy of individual ICD-10 codes used for HVA clinical documentation. Patients identified from the Tricare beneficiary population health registry database who were also identified in the IT database (*n* = 1,059) were excluded from this random sample. Chart review revealed that most of these patients 76.0% (*n* = 114) did not have Hymenoptera-venom anaphylaxis (Figure 3b). Of the 114 patients who were not diagnosed with Hymenoptera-venom anaphylaxis, 4 had large local reactions, 17 had diffuse cutaneous reactions, 40 had localized swelling, 26 had reactions due to non-Hymenoptera insects, 1 had a delayed reaction, and 26 had no documentation of any insect reaction in their medical records.

Of the 150 patients with one or more of the ICD-10 codes who underwent chart review, 24% (*n* = 36) were diagnosed with Hymenoptera-venom anaphylaxis. Of the 36 patients with confirmed Hymenoptera-venom anaphylaxis, 16.7% (*n* = 6) had FA-venom anaphylaxis only, 66.7% (*n* = 24) had FH-venom anaphylaxis only, and 16.7% (*n* = 6) had both FA- and FH-venom anaphylaxis. 33.3% (4/12) of patients diagnosed with FA-venom anaphylaxis were treated with FA WBE-IT and 36.7% (11/30) of patients diagnosed with FH-venom anaphylaxis were treated with IT. All patients found to be on IT treatment through the population health registry that were not found in IT prescription database were found to have obtained IT treatments through private sector care, demonstrating the robustness of the IT dataset.

### Hymenoptera hypersensitivity prevalence and incidence estimates

There were 6,147 patients with an HVA ICD-10 code from the Tricare beneficiary population health registry database after excluding the 1,059 patients who were also in the IT prescription database. Of the 6,147 patients, 492 patients were estimated to have a FA-venom anaphylaxis, assuming a true positive rate of 8%. An 8% true positive rate, was determined based on identifying 12 patients with FA-venom anaphylaxis of the 150 randomly selected patients with an HVA ICD-10 code who underwent chart review. Of the 6,147 patients, 1,229 patients were estimated to have a FH-venom anaphylaxis, assuming a true positive rate of 20%. A 20% true positive rate was determined based on identifying 30 patients with FH-venom anaphylaxis of the 150 randomly selected patients with an HVA ICD-10 code who underwent chart review. We then estimated the overall prevalence of FA- and FH- venom anaphylaxis by combining the data from IT prescription database with the Tricare beneficiary population health registry databases (Figures 3c,d). Overall, 2,371 of 2,835,948 patients enrolled in direct care were estimated to have one or more FH-venom anaphylaxis, for a prevalence of 0.083% (8.3 per 10,000 individuals). Further, 1,370 of 2,835,948 patients enrolled in direct care were estimated to have FA-venom anaphylaxis, for a prevalence of 0.048% (4.8 per 10,000 individuals). Notably, the FA-venom anaphylaxis prevalence was 0.085% (8.5 per 10,000 individuals) when only considering the fourteen states that are colonized by FAs.

In the calendar year 2023, there were 187 new IT prescriptions for 176 Tricare beneficiaries in direct care from the IT prescription database. Of those 187, 72 were for FA per 2,835,948 persons and 115 were for FH per 2,835,948 persons. Eleven patients were ordered both FA and FH prescriptions. Of the 115 FH prescriptions, there were 85 W, 82 MV, 75 HB, 7 WFH, 10 YJ, 0 YH.

### Tricare beneficiaries with SM or MMAS and FA-venom anaphylaxis

**Figure 4:**
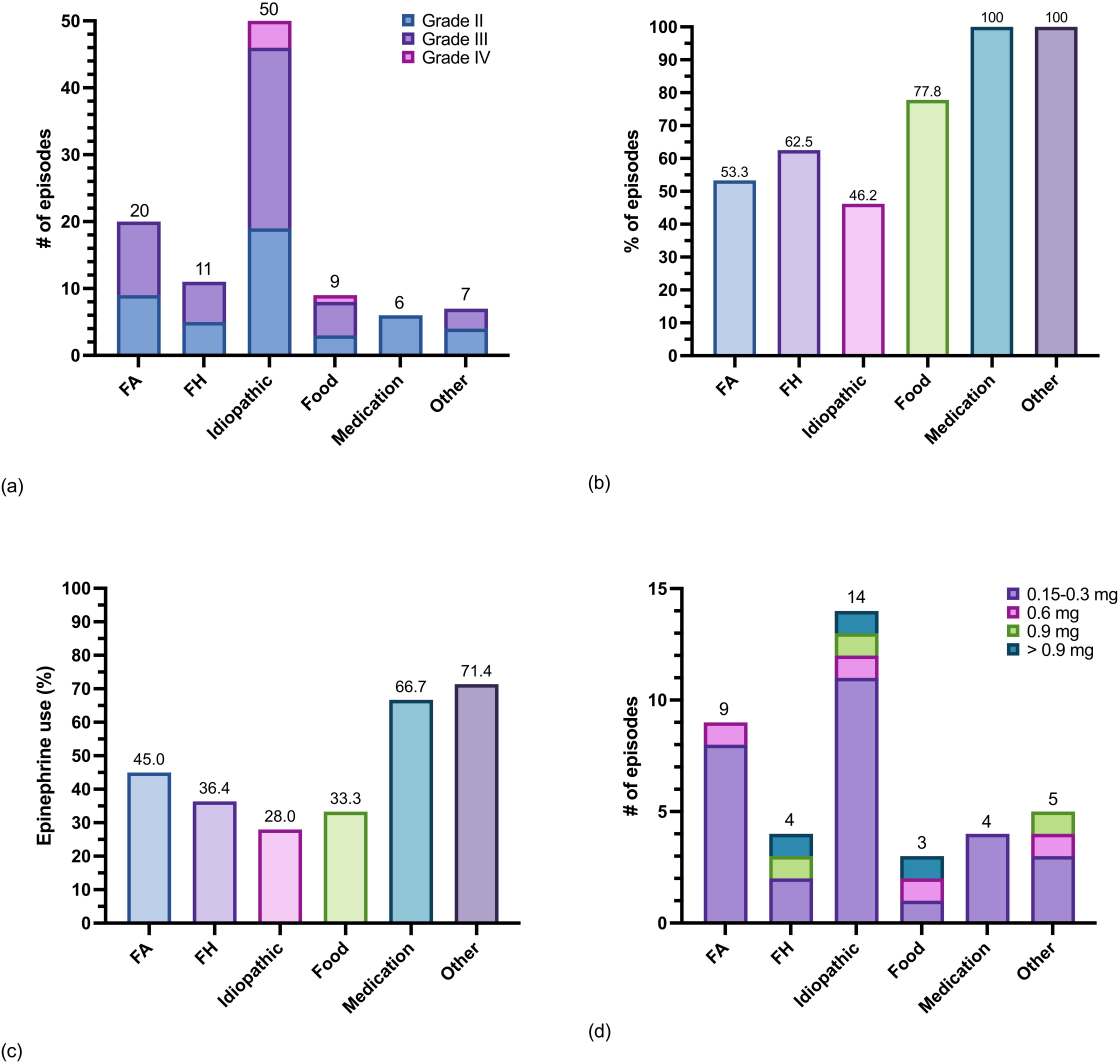
Triggers of anaphylaxis and epinephrine use in 43 patients with SM and MMAS. **(a)** Counts of episodes of anaphylaxis grouped by trigger and shaded by severity (Supplementary Table 2), **(b)** percent of anaphylaxis episodes grouped by trigger that were evaluated at a healthcare facility and diagnosed as anaphylaxis, **(c)** percent of anaphylaxis episodes grouped by trigger treated with epinephrine by either the patient or a healthcare provider, and **(d)** total epinephrine dose administered per episode by trigger. Note: one patient with SM had anaphylaxis after receiving the Measles Mumps and Rubella vaccine (counted in “other” category).

A total of 97 patients, including 96 with SM and one with MMAS, from a previously described cohort, were analyzed for anaphylaxis triggers, severity, sensitization evaluations, and epinephrine treatment patterns (40, 50). Most patients had indolent or presumed indolent SM (ISM) 84.5% (*n* = 89), no patients had smoldering SM, and 7.2% (*n* = 7) had an advanced SM subtype (aggressive SM, SM with an associated myeloid neoplasm, or mast cell leukemia). Most patients 84.5% (*n* = 82) had *KIT* p.D816 V detected in peripheral blood, bone marrow aspirate, or both (Table 1). Mean BST values and mean *KIT* p.D816 V variant allele frequencies were significantly lower in patients with compared to those without a history of anaphylaxis (Supplementary Figure 2). There were 27 patients with bone marrow mastocytosis (BMM), a subvariant of ISM. A total of 44.3% (*n* = 43) of patients with SM or MMAS had a history of anaphylaxis and there were 103 episodes of anaphylaxis documented. Of the 103 total episodes of anaphylaxis, 44.7% (*n* = 46) were grade II, 50.5% (*n* = 52) were grade III, and 4.8% (*n* = 5) were grade IV. Anaphylaxis episodes in 37.9% (*n* = 39) were treated with epinephrine and of those 39, 25.6% (*n* = 10) required treatment with a dose higher than 0.3 mg (Figure 4a).

**Table 1:** Demographics.

Characteristic	SM Population (*n* = 97)	SM with Anaphylaxis (*n* = 43)
Age (years), median (IQR)	47.0 (37.0–60.0)	51.0 (36.0–60.0)
Sex		
Female	38.1% (37/97)	18.6% (8/43)
Male	61.9% (60/97)	81.4% (35/43)
Mastocytosis diagnosis		
Presumed indolent systemic mastocytosis^a^	4.1% (4/97)	2.3% (1/43)
Indolent systemic mastocytosis	87.6% (85/97)	93.0% (40/43)
Bone Marrow Mastocytosis	31.8% (27/85)	62.5% (25/40)
Aggressive systemic mastocytosis	3.1% (3/97)	0.0% (0/43)
SM with an associated myeloid neoplasm	3.1% (3/97)	0.0% (0/43)
Mast cell leukemia	1.0% (1/97)	2.3% (1/43)
Non-SM clonal mast cell disorder		
Monoclonal mast cell activation syndrome	1.0% (1/97)	2.3% (1/43)
History of monomorphic MPCM	63.9% (62/97)	34.9% (15/43)
Lab markers		
*KIT* p.D816 V PB or BM—positive/total (%)	84.5% (82/97)	83.7% (36/43)
*KIT* p.D816 V VAF %, median (IQR) (*n* = 62 | *n* = 31)	0.09 (0.04–0.39)	0.06 (0.04–0.25)
Basal serum tryptase (BST), median (IQR)	20.8 (13.8–38.3)	15.5 (11.7–25.6)
Anaphylaxis trigger		
Hymenoptera venom	—–	51.2% (22/43)
Idiopathic	—–	44.2% (19/43)
Food	—–	14.0% (6/43)
Medications	—–	11.6% (5/43)
Allergen immunotherapy	—–	9.3% (4/43)
Other	—–	4.7% (2/43)
Beta-blocker use	—–	4.7% (2/43)
ACE-inhibitor use	—–	7.0% (3/43)

^a^ Presumed indolent systemic mastocytosis reflects patients had *KIT* p.D816 V detected in peripheral blood but did not have a bone marrow biopsy to evaluate the WHO major and minor criteria.

Of the 43 patients with a history of anaphylaxis, a Hymenoptera envenomation trigger was identified in 51.2% (*n* = 22) of patients. More patients had anaphylaxis triggered by FA 32.6% (*n* = 14) compared to all FH combined 25.6% (*n* = 11) (Supplementary Table 4). Of the 103 episodes of anaphylaxis total, FA was the second most common trigger of anaphylaxis in patients with SM or MMAS overall 19.4% (*n* = 20) after idiopathic anaphylaxis 48.5% (*n* = 50) (Figure 4b). Regarding severity of each FA- venom anaphylaxis episode, 55.0% (*n* = 11) were grade III and 45.0% (*n* = 9) were grade II. Nearly half of all episodes of FA-venom anaphylaxis were not initially diagnosed as anaphylaxis or treated with epinephrine (Figures 4c,d). Thirteen of 14 patients with a clinical history of FA-venom anaphylaxis had evidence of sensitization by skin testing or serum specific-IgE testing. Ten of 11 patients with a clinical history of FH-venom anaphylaxis had evidence of sensitization by serum specific-IgE and/or skin testing. Most patients with FA-venom anaphylaxis were treated with FA WBE-IT (Supplementary Table 4).

## Discussion

Red and black FAs, native to South America, invaded the U.S. in the 1900s, now reside in 14 states, and continue to invade other locations such as China, Taiwan, Australia, New Zealand, Trinidad, and Italy (51, 52). FAs are predicted to spread to a much broader area by 2050, including in Europe and Asia, in part, due to climate change (53, 54). FAs may be more commonly encountered in regions they invade compared to other stinging Hymenoptera as these areas often lack the natural predators present in South America. In fact, multiple stings from one or more FA may occur per individual exposure, lifetime exposure rates approach 100%, and sensitization rates are high relative to FH in colonized areas (11). The prevalence of anaphylaxis to red and black FA venom is likely to surpass other stinging Hymenoptera in many parts of the world.

Accordingly, we have shown that FA WBE-IT treatments prescribed by allergists are now the most prescribed individual IT treatment among all Tricare beneficiaries despite many states in the U.S. having no FAs. FA WBE-IT was most often prescribed as a monotherapy without FH IT since patients with ant stings are usually not tested for FH sensitization. Patients living in a colonized area along with identification of a FA mound or an ant allows for targeted testing of FA. Nonetheless, we found that patients treated with FA WBE-IT may also be treated with W IT and MV IT, and to a lesser extent HB IT. Interestingly, FA and HB are more closely related to each other than either are to species in the Vespidae family (1). However, more venom proteins are shared between FA and Vespidae family species and cross reactivity between venoms from both groups has been reported (55).

To our knowledge, we are the first to present a detailed estimate of the prevalence of FA-venom anaphylaxis in the U.S. general population and in patients with SM. To accomplish this, we combined data from two unique databases to estimate the prevalence of FA- and FH-venom anaphylaxis. The centralized beneficiary IT database allowed us to identify patients prescribed IT by allergists and the Tricare beneficiary population health registry database allowed us to identify patients assigned one or more of five ICD-10 codes frequently used to document HVA in medical records of patients that were either not prescribed IT or had IT ordered in private sector care. We showed that the ICD-10 code true positive rate for Hymenoptera-venom anaphylaxis is low based on a random sample of 150 patients. Surprisingly, the estimated prevalence of FA-venom anaphylaxis in the fourteen states colonized by FA was 0.085% and greater than the prevalence of all other FH combined. Additionally, the prevalence of FH-venom anaphylaxis we presented of 0.083% was lower than prior studies that ranged from 0.17%–3.3%. (41–48)

SM has been linked to fatal anaphylaxis triggered by FH (25, 33). It is not currently known what percent of the 60–100 patients that die each year in the U.S. from FH venom anaphylaxis have SM, although it is likely to be high. Only one large series of autopsies has been reported on 100 patients who died from FH stings, but this was before SM diagnostic testing was available (23). In 1989, there were 32 reported deaths linked to FA- venom anaphylaxis (20), but SM was not assessed in any of those deaths and no studies assessing mortality from FA stings have been published since then. Here we have shown that FA is a more common trigger for grade II or III anaphylaxis than all other FH combined among a cohort of Tricare beneficiaries with SM and MMAS. In patients with SM and a history of anaphylaxis, 31.0% had FA as a trigger. Unfortunately, nearly 50.0% of episodes of FA- venom anaphylaxis were not initially recognized as anaphylaxis or treated with epinephrine. Higher doses of epinephrine were needed to treat anaphylaxis in many patients with SM regardless of the anaphylaxis trigger suggesting these patients would benefit from having two or more epinephrine autoinjectors available. An epinephrine dose of 0.3 mg is now regarded as being inadequate even among many patients without SM (56).

There are several limitations to our study. First, heterogenous exposure to FA and FH stings in the community may impact prevalence estimates. Second, IT might rarely be ordered for patients with sensitization, frequent exposure, and large local reactions without a history of anaphylaxis and this might lead to an overestimate of Hymenoptera venom anaphylaxis. Third, the portion of Tricare beneficiaries enrolled in direct care living in some states colonized by FAs may be more than the general population and this may lead to an overestimate of FA-venom anaphylaxis prevalence in the U.S. However, it is important to note that the proportion of Tricare beneficiaries relative to the general population was decreased in 5 of 14 FA-colonized states including Arizona, California, Florida, Louisiana, and Tennessee (Supplementary Table 5). Fourth, the Hymenoptera-venom anaphylaxis prevalence may be underestimated due to ADSMs being less likely to seek diagnosis and treatment given potential career implications. Lastly, the prevalence of Hymenoptera-venom anaphylaxis that we report may be influenced by the fact that ADSMs, who made up 36% of our study population, may have a higher occupational exposure to Hymenoptera stings.

In summary, the burden of FA-venom anaphylaxis in the general population and among patients with SM has surpassed all other FH-venom anaphylaxis combined in many areas of the U.S. colonized by FAs and FA-venom anaphylaxis is now the most treated HVA among all active Tricare beneficiaries. This is alarming since climate change is expected to substantially expand FA habitats. This has additional implications for Europe and Asia where FAs have recently colonized. Anaphylaxis triggered by FAs in patients with SM is often mistaken for another diagnosis and not treated promptly with epinephrine. Future research should seek to estimate the number of deaths attributed to HVA, and FAs in particular, with a focus on how many of these deaths are due, in part, to underlying SM.

## Data Availability

The raw data supporting the conclusions of this article will be made available by the authors, without undue reservation.
