# Supplementary material for: Fire ant-venom anaphylaxis prevalence in the general population and patients with systemic mastocytosis
Source: Front Allergy. 2025 Mar 31;6:1570123. doi: 10.3389/falgy.2025.1570123 (PMC11994729; doi:10.3389/falgy.2025.1570123)
Supplement: Supplementary file 1 [file Datasheet1.docx]

**Supplemental Table 1.** ICD-10 Codes Specific to Hymenoptera Venom Allergy†

| **ICD-10** | **Specific Description** |
| --- | --- |
| **Z91.038** | Other insect allergy status |
| **Z91.030** | Bee allergy status |
| **T63.44** | Toxic effect of venom of bees |
| **T63.45** | Toxic effect of venom of hornets |
| **T63.46** | Toxic effect of venom of wasps |

†ICD-10 codes used to identify patients in the Tricare beneficiary population health registry database.

The five ICD-10 codes for venom allergy correspond to the codes used in the Schuler et al study.^1^

**Supplemental Table 2.** Ring and Messmer Anaphylaxis Severity Scale†

| **Grade** | **Symptoms** |
| --- | --- |
| **Grade I** | Cutaneous symptoms +/- mild fever reaction |
| **Grade II** | Non-life-threatening reaction – Cardiovascular (tachycardia, hypotension), Gastrointestinal symptoms (nausea), Respiratory symptoms |
| **Grade III** | Shock, life-threatening smooth muscle spasm (bronchi, etc.) |
| **Grade IV** | Cardiac and/or respiratory arrest |

†Adapted from Ring J, Messmer K.^2^

**Supplemental Table 3.** Imported Fire Ant Quarantine Dates†

| **State** | **Quarantine Dates** |
| --- | --- |
| **Alabama** | 1918 (Mobile) – 1988 (Jackson) |
| **Arkansas** | 1958 (Union) – 2022 (Franklin) |
| **California** | 1999 (Riverside) – 2022 (Orange) |
| **Florida** | 1939 (Escambia) – 1976 (Broward) |
| **Georgia** | 1958 (Crisp) – 2003 (Rabun) |
| **Louisiana** | 1953 (East Baton Rouge) – Claiborne (1973) |
| **Mississippi** | 1939 (George) – 1996 (Coahoma) |
| **New Mexico** | 2000 (Dona Ana) |
| **North Carolina** | 1971 (Brunswick) – 2021 (Alamance) |
| **Oklahoma** | 1988 (Bryan) – 2024 (Haskell) |
| **Puerto Rico** | 1982 (Mayaguez) – 1983 (Adjuntas) |
| **South Carolina** | 1958 (Charleston) – 1999 (Cherokee) |
| **Tennessee** | 1990 (Hardin) – 2024 (Robertson) |
| **Texas** | 1958 (Hardin) – 2016 (Mills) |
| **Virginia** | 2009 (James City) – 2023 (Charlotte) |

†Data extracted from U.S. Department of Agriculture (USDA)

“Imported Fire Ant Federal Quarantine” Interactive Map.^3^ A FA

quarantine area is added if colonies have been present for 3 consecutive

years. Date ranges above include the date and associated county in

parentheses.

**Supplemental Table 4**. Hymenoptera-Venom Anaphylaxis in Systemic Mastocytosis: Sensitization Patterns

| **Characteristic** | **Result** |
| --- | --- |
| **Clinical History of Venom Anaphylaxis** |  |
| Fire ant | 32.6% (14/43) |
| Flying Hymenoptera | 25.6% (11/43) |
| **Sensitization by Serum and/or SPT** |  |
| Fire ant sensitization | 92.3% (13/14) |
| Flying Hymenoptera sensitization | 90.9% (10/11) |
| Yellow jacket sensitization | 81.8% (9/11) |
| Paper wasp sensitization | 72.7% (8/11) |
| White-faced hornet sensitization | 63.6% (7/11) |
| Honeybee sensitization | 36.4% (4/11) |
| Yellow hornet sensitization | 27.3% (3/11) |
| **Serum Venom Sensitization** | **< 0.10 \| 0.10 – 0.34 \| ≥ 0.35 (n)** |
| Fire ant IgE (n = 13) | 7.7% (1/13) \| 7.7% (1/13) \| 84.6% (11/13) |
| Yellow jacket IgE (n = 11) | 27.3% (3/11) \| 27.3% (3/11) \| 45.5% (5/11) |
| Paper wasp IgE (n = 11) | 27.3% (3/11) \| 18.2% (2/11) \| 54.5% (6/11) |
| White-faced hornet (n = 11) | 45.5% (5/11) \| 18.2% (2/11) \| 36.4% (4/11) |
| Honeybee IgE (n = 11) | 72.7% (8/11) \| 9.1% (1/11) \| 18.2% (2/11) |
| Yellow hornet (n = 11) | 72.7% (8/11) \| 0.0% (0/11) \| 27.3% (3/11) |
| **SPT Sensitization, % positive/total tested** |  |
| Fire ant (n = 1) | 100.0% (1/1) |
| Yellow jacket (n = 2) | 100.0% (2/2) |
| Paper wasp (n = 2) | 50.0% (1/2) |
| White-faced hornet (n = 3) | 66.7% (2/3) |
| Honeybee (n = 3) | 33.3% (1/3) |
| Yellow hornet (n = 3) | 33.3% (1/3) |
| **Discordance between Serum and SPT†** |  |
| Flying Hymenoptera (n = 4) | 75.0% (3/4) |
| **History of Venom Immunotherapy** | 61.9% (13/21) |
| Fire ant (n = 13) | 69.2% (9/14) |
| Flying Hymenoptera (n = 11) | 63.6% (7/11) |
| **Currently on Venom Immunotherapy** | 57.1% (12/21) |
| Fire ant (n = 13) | 61.5% (8/14) |
| Flying Hymenoptera (n = 11) | 63.6% (7/11) |

†4 patients had both serum and skin prick test (SPT) testing to Hymenoptera venom. All 14 patients with FA-triggered anaphylaxis had either fire ant (FA) serum or skin testing; none had both.

**Supplemental Table 5.** Relative Proportions of Current Tricare Beneficiaries in Direct Care by State

| **State** | **FA Endemic State (Y/N)** | **Current Beneficiaries** | **State Population (2023)†** | **Beneficiary Ratio‡** | **Beneficiaries Overrepresented (Y/N)** |
| --- | --- | --- | --- | --- | --- |
| **VA** | Y | 284,496 | 8,715,698 | 4.25138931 | Y |
| **NC** | Y | 168,208 | 10,835,491 | 2.02187795 | Y |
| **NM** | Y | 32,584 | 2,114,371 | 2.0071512 | Y |
| **GA** | Y | 138,221 | 11,029,227 | 1.63224686 | Y |
| **SC** | Y | 63,271 | 5,373,555 | 1.53355687 | Y |
| **TX** | Y | 317,979 | 30,503,301 | 1.35771459 | Y |
| **MS** | Y | 30,135 | 2,939,690 | 1.3351392 | Y |
| **OK** | Y | 41,536 | 4,053,824 | 1.33449413 | Y |
| **AL** | Y | 44,501 | 5,108,468 | 1.13458223 | Y |
| **FL** | Y | 167,106 | 22,610,726 | 0.96257465 | N |
| **LA** | Y | 31,889 | 4,573,749 | 0.90808282 | N |
| **CA** | Y | 241,465 | 38,965,193 | 0.80711281 | N |
| **TN** | Y | 34,463 | 7,126,489 | 0.62984596 | N |
| **AR** | Y | 11,760 | 3,067,732 | 0.49928301 | N |
| **AK** | N | 53,284 | 733,406 | 9.46257307 | Y |
| **HI** | N | 93,452 | 1,435,138 | 8.48109292 | Y |
| **DC** | N | 16,451 | 678,972 | 3.15571172 | Y |
| **ND** | N | 14,769 | 783,926 | 2.45376466 | Y |
| **WA** | N | 141,558 | 7,812,880 | 2.35982691 | Y |
| **KS** | N | 50,227 | 2,940,546 | 2.22467281 | Y |
| **CO** | N | 97,240 | 5,877,610 | 2.15477074 | Y |
| **MD** | N | 95,295 | 6,180,253 | 2.00826373 | Y |
| **NV** | N | 46,738 | 3,194,176 | 1.90575982 | Y |
| **WY** | N | 8,136 | 584,057 | 1.81431458 | Y |
| **KY** | N | 45,031 | 4,526,154 | 1.2958035 | Y |
| **SD** | N | 8,832 | 919,318 | 1.25126755 | Y |
| **NE** | N | 18,632 | 1,978,379 | 1.22661103 | Y |
| **DE** | N | 8,262 | 1,031,890 | 1.04281837 | Y |
| **MT** | N | 8,449 | 1,132,812 | 0.97141399 | N |
| **AZ** | N | 47,121 | 7,431,344 | 0.82585541 | N |

**Supplemental Table 5 (cont.).** Relative Proportions of Current Tricare Beneficiaries in Direct Care by State

| **State** | **FA Endemic State (Y/N)** | **Current Beneficiaries** | **State Population (2023)†** | **Beneficiary Ratio‡** | **Beneficiaries Overrepresented (Y/N)** |
| --- | --- | --- | --- | --- | --- |
| **MO** | N | 31,730 | 6,196,156 | 0.66696741 | N |
| **RI** | N | 5,171 | 1,095,962 | 0.61451987 | N |
| **ID** | N | 8,174 | 1,964,726 | 0.54186304 | N |
| **UT** | N | 14,208 | 3,417,734 | 0.54144154 | N |
| **OH** | N | 33,094 | 11,785,935 | 0.36571444 | N |
| **CT** | N | 8,204 | 3,617,176 | 0.29540164 | N |
| **IL** | N | 28,053 | 12,549,689 | 0.29114091 | N |
| **NY** | N | 37,926 | 19,571,216 | 0.25239231 | N |
| **NJ** | N | 16,221 | 9,290,841 | 0.22739425 | N |
| **NH** | N | 1,708 | 1,402,054 | 0.15866459 | N |
| **PA** | N | 11,866 | 12,961,683 | 0.11923392 | N |
| **MA** | N | 6,343 | 7,001,399 | 0.11799586 | N |
| **ME** | N | 1,048 | 1,395,722 | 0.09779558 | N |
| **IA** | N | 1,603 | 3,207,004 | 0.06510149 | N |
| **WV** | N | 759 | 1,770,071 | 0.05584804 | N |
| **OR** | N | 1,112 | 4,233,358 | 0.03421186 | N |
| **WI** | N | 959 | 5,910,955 | 0.02113089 | N |
| **VT** | N | 102 | 647,464 | 0.0205183 | N |
| **MI** | N | 945 | 10,037,261 | 0.01226234 | N |
| **IN** | N | 629 | 6,862,199 | 0.01193835 | N |
| **MN** | N | 501 | 5,737,915 | 0.0113721 | N |

**†**Includes the estimated state population in 2023 per the United States Census Bureau.^4^

‡Beneficiary ratio represents the proportion of current Tricare beneficiaries by state compared to the state population. The beneficiary ratio was determined

using the following formula = (Tricare current beneficiaries in specific state/total Tricare beneficiaries) / (estimated state population in 2023/estimated

U.S. population in 2023). The total Tricare beneficiaries included the 50 states and D.C. (2,571,447), and the estimated U.S. population in 2023 included the

50 states and D.C. (334,914,895). A ratio greater than 1 indicates there was an increased proportion of Tricare beneficiaries living in a particular state relative

to the general population. A ratio less than 1 indicates there was a decreased proportion of Tricare beneficiaries living in a particular state relative to the general

population. A ratio of 1 indicates there was an equal proportion of Tricare beneficiaries living in a particular state relative to the general population.

**Supplemental Figure Legend**

**Supplemental Figure 1. Analysis of Two Separate Databases to Estimate Occurrence of Hymenoptera-venom anaphylaxis among Tricare beneficiaries** (a) Immunotherapy database: Tricare beneficiaries ordered flying Hymenoptera (FH) venom immunotherapy (IT) and fire ant (FA) whole-body extract IT (WBE-IT). The beneficiary immunotherapy prescription database was queried for all IT extract orders from 1990-2023. Patients treated only with aeroallergen IT and not venom IT or FA WBE-IT were excluded. Additionally, there were 3,655 patients ordered FH venom IT and 1,877 patients ordered FA WBE-IT between 1990-2023 who were not current beneficiaries and were excluded. There were 1,142 current beneficiaries who had one or more IT prescription(s) ordered for FH venom and 878 current beneficiaries who had FA WBE-IT ordered (Figure 1a) who were included in the prevalence calculation. (b) Health registry database: Identification and analysis of current Tricare beneficiaries with Hymenoptera-venom anaphylaxis not identified in part a. There were 7,206 patients identified who had one or more Hymenoptera-venom allergy ICD-10 code (Supplemental Table 1). 1,059 were excluded since they were already identified and counted from the immunotherapy database in part a. Physician-chart review of a sample of 150 patients randomly selected from the remaining 6,147 patients demonstrated that 24% had Hymenoptera-venom anaphylaxis. *246 beneficiaries were estimated to have both FH-venom anaphylaxis and FA-venom anaphylaxis based on 4% (n = 6) of patients in the random sample having both FH- and FA-venom triggers of anaphylaxis.

**Supplemental Figure 2 Disease Burden Biomarkers in Patients with SM.** (a) Continuous scatter plots comparing BST means and distributions in patients with SM with and without a history of anaphylaxis. (b) Continuous scatter plots comparing *KIT* p.D816V variant allele frequency (VAF) means and distributions in patients with SM with and without a history of anaphylaxis. Mann-Whitney test was used (p-value) to compare means of the 2 groups.

**Supplemental Methods**

*Hymenoptera-Venom Anaphylaxis ICD-10 Code Accuracy Analysis*

The accuracy of five ICD-10 codes (Supplemental Table 1) in identifying a patient with Hymenoptera-venom anaphylaxis was assessed by individual chart review of a random sample of patients for each specific code. Patients known to be on IT treatment as indicated by the data obtained from the beneficiary immunotherapy prescription database were excluded from this accuracy analysis to estimate the additional patients with Hymenoptera-venom anaphylaxis and overall prevalence. Patients were separated according to their most recent HVA ICD-10 codes. A random sample of patients with each ICD-10 code was obtained utilizing the randomization function “Rand()” in Microsoft Excel. The column containing the “Rand()” function was then sorted in ascending order. The first 30 patients for each ICD-10 code were selected for chart review. A patient was determined to have one or more stinging Hymenoptera hypersensitivity reaction according to NIAID criteria and utilizing practice parameter guidelines.^5–8^ In brief, patients with stinging Hymenoptera hypersensitivity were required to have a clinical presentation of an immediate generalized hypersensitivity reaction to FA, HB, YJ, WFH, YH, and/or W along with sensitization by skin testing and/or serum specific-IgE testing. A positive FH venom skin test required either an a) epicutaneous (prick) skin test to FH venom at a concentration of 1.0 mcg/mL or b) intradermal skin test to venom at a concentration of ≤ 1.0 mcg/mL. A positive FA venom skin test required either an a) epicutaneous skin test to FA WBE at a concentration of ≤ 1:100 wt/vol or b) intradermal skin test to FA WBE at a concentration ≤ 1:1,000 wt/vol. A wheal diameter 3 mm or greater than the negative control on epicutaneous or intradermal testing was required for positive FH and FA skin tests. Venom-specific IgE ≥0.1 kU/L on ImmunoCAP was required for a positive serum assay.

*Systemic Mastocytosis and Monoclonal Mast Cell Activation Diagnostic Criteria*

Patients were diagnosed with systemic mastocytosis (SM) utilizing the World Health Organization (WHO) 2017 consensus criteria by fulfilling the major and one minor criterion or by fulfilling three minor criteria.^9^ The major criterion is met when two or more dense aggregates comprised of 15 or more mast cells are seen in bone marrow tissue sections. The four minor criteria include greater than 25% of mast cells demonstrating spindled morphology in core needle biopsy sections or atypical morphology on bone marrow aspirate smears; aberrant expression of CD25 and/or CD2, by immunohistochemistry or flow cytometry; detection of the *KIT* activating mutation at codon 816, in either peripheral blood or bone marrow aspirate; and a basal serum tryptase ≥ 20 ng/mL in the absence of another myeloid neoplasm.

The diagnosis of monoclonal mast cell activation (MMAS) was assigned to any patient who did not meet the WHO major criteria for SM, met 1 or 2 WHO minor criteria for SM, and at least one of the minor criteria that were met had to include either CD25 expression on mast cells or detection of *KIT* p.D816V in peripheral blood or bone marrow.^10^

*Anaphylaxis in patients with SM and MMAS*

A patient with SM or MMAS was determined to have anaphylaxis according to NIAID criteria.^8^

**References**

1     Schuler CF, Volertas S, Khokhar D, *et al.* Prevalence of mastocytosis and Hymenoptera venom allergy in the United States. *J Allergy Clin Immunol* 2021; **148**: 1316–23.

2     Ring J, Messmer K. Incidence and severity of anaphylactoid reactions to colloid volume substitutes. *Lancet* 1977; **1**: 466–9.

3     USDA APHIS | Imported Fire Ants (IFA) Interactive Map. Imported Fire Ant Federal Quarantine. https://www.aphis.usda.gov/aphis/maps/plant-health/ifa-quarantine-mapping (accessed March 24, 2024).

4     United States Census Bureau. State Population Totals: 2020-2023. State Population Totals and Components of Change: 2020-2023. 2023; published online Dec 18. https://www.census.gov/data/tables/time-series/demo/popest/2020s-state-total.html (accessed April 21, 2024).

5     Golden DBK, Wang J, Waserman S, *et al.* Anaphylaxis: A 2023 practice parameter update. *Ann Allergy Asthma Immunol* 2024; **132**: 124–76.

6     Golden DBK, Moffitt J, Nicklas RA, *et al.* Stinging insect hypersensitivity: a practice parameter update 2011. *J Allergy Clin Immunol* 2011; **127**: 852-4.e1.

7     Golden DBK, Demain J, Freeman T, *et al.* Stinging insect hypersensitivity: A practice parameter update 2016. *Ann Allergy Asthma Immunol* 2017; **118**: 28–54.

8     Sampson HA, Muñoz-Furlong A, Campbell RL, *et al.* Second symposium on the definition and management of anaphylaxis: summary report--Second National Institute of Allergy and Infectious Disease/Food Allergy and Anaphylaxis Network symposium. *J Allergy Clin Immunol* 2006; **117**: 391–7.

9     WHO Classification of Tumours Editorial Board, Campo E, Harris NL, *et al.* WHO Classification of Tumours of Haematopoietic and Lymphoid Tissues (Medicine), Revised. World Health Organization, 2017.

10    Valent P, Hartmann K, Bonadonna P, *et al.* Global Classification of Mast Cell Activation Disorders: An ICD-10-CM-Adjusted Proposal of the ECNM-AIM Consortium. *J Allergy Clin Immunol Pract* 2022; **10**: 1941–50.
