# Supplementary figures and images for: Fire ant-venom anaphylaxis prevalence in the general population and patients with systemic mastocytosis
Source: Front Allergy. 2025 Mar 31;6:1570123. doi: 10.3389/falgy.2025.1570123 (PMC11994729; doi:10.3389/falgy.2025.1570123)

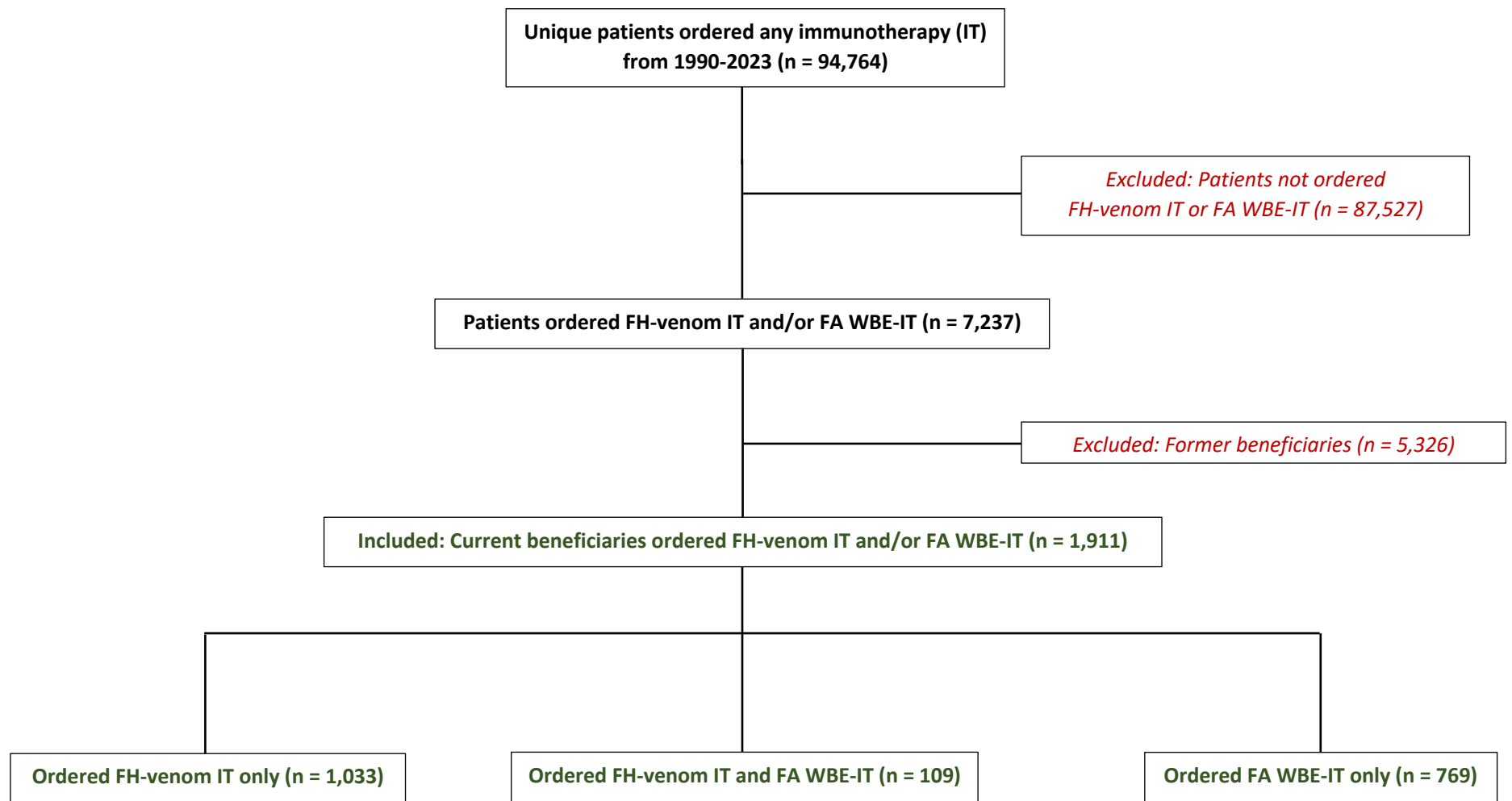

Supplemental Figure 1a

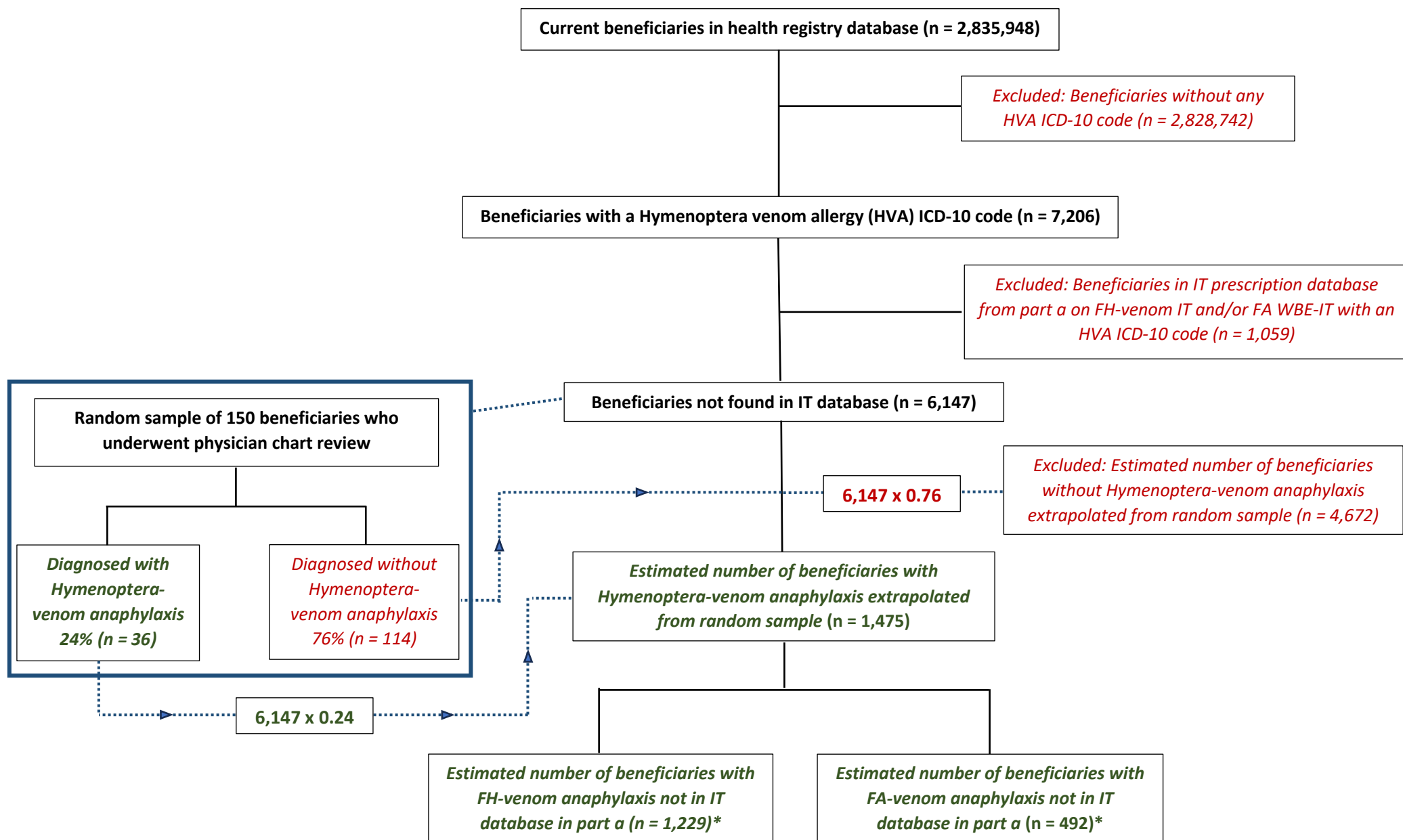

Supplemental Figure 1b

Supplement: Supplementary file 2 [file Image1.pdf]

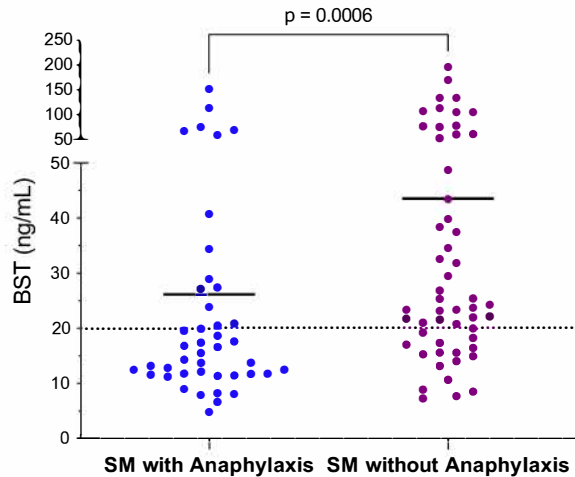

(a)

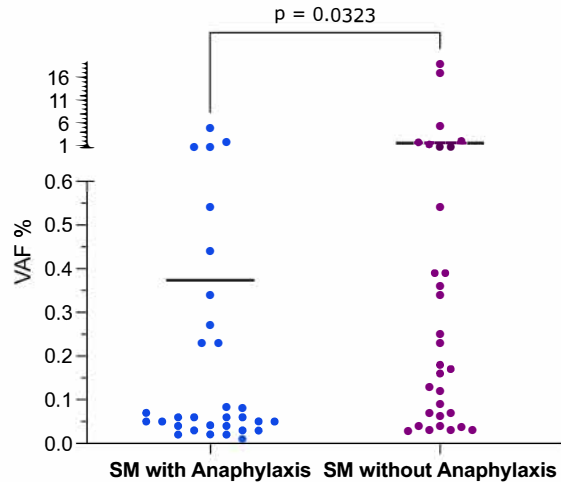

(b)

**Supplementary Figure 2**

Supplement: Supplementary file 3 [file Image2.pdf]
